# ICD-11 Personality Disorders: A Psychodynamic Perspective on Personality Functioning

**DOI:** 10.3389/fpsyt.2021.654026

**Published:** 2021-04-16

**Authors:** Victor Blüml, Stephan Doering

**Affiliations:** Department of Psychoanalysis and Psychotherapy, Medical University of Vienna, Vienna, Austria

**Keywords:** personality disorder classification, personality functioning, self and interpersonal functioning, psychoanalysis, psychodynamic, object relations theory, OPD, ICD-11

## Abstract

The new ICD-11 introduces a fully dimensional classification of personality disorders representing a fundamental change in personality disorder diagnosis with major implications for clinical practice and research. The new system centers on the evaluation of the severity of impairment in the areas of self and interpersonal functioning. This focus on personality functioning converges with long-standing psychoanalytic/psychodynamic conceptualizations of personality pathology. In a detailed conceptual analysis and review of existing empirical data, points of convergence and notable differences between major exponents of the psychodynamic tradition—object relations theory as developed by Kernberg et al. and the Operationalized Psychodynamic Diagnosis—and the ICD-11 system are critically discussed. Personality functioning can be considered to be the current “common ground” for the assessment of personality disorders and constitutes a considerable step forward in making personality disorder diagnosis both clinically meaningful and suitable for research purposes.

## Introduction

The upcoming 11th revision of the ICD includes “the most radical change in the personality disorders classification history” (1). Arguably the biggest change is the move from a categorical classification of personality disorders to a fully dimensional system (2). The “traditional” categorical classification system has been the subject of decades-long critique and its deficiencies and shortcomings are well-known (3). A move toward a dimensional system was already planned for the last revision of the DSM, but in the last instance the old categorical model was retained in the main corpus and the newly developed “Alternative Model for Personality Disorders” (AMPD) was placed in section III reserved for emerging models and measures (4). The AMPD provides a hybrid dimensional-categorical model with an evaluation of core personality functioning and five broad areas of pathological personality traits as well as identifying six specific personality disorder types (5, 6). Meanwhile, the ICD-11 originally planned to abandon all specific personality disorders, but after intense criticism from several personality disorder expert organizations a special “borderline pattern descriptor” was included in the final version (1, 7). However, at its core the new ICD-11 presents the first official version of a purely dimensional personality disorder classification with major implications for clinical practice and research.

One of the key issues is the new focus on personality functioning as the essence of personality disorder classification. The ICD-11 fundamentally defines personality disorders by “problems in functioning of aspects of the self (e.g., identity, self-worth, accuracy of self-view, self-direction), and/or interpersonal dysfunction (e.g., ability to develop and maintain close and mutually satisfying relationships, ability to understand others' perspectives and to manage conflict in relationships)” (8). This definition closely resembles Criterion A of the AMPD of DSM-5, which equally posits personality functioning as the sine qua non for the diagnosis of a personality disorder in general. Personality functioning in the AMPD is specified as intrapersonal (self) and interpersonal (other) functioning, which are in turn each subdivided into 2 aspects, identity and self-direction for self-functioning, as well as empathy and intimacy for interpersonal functioning (5).

Impairment in personality functioning constitutes the core component of personality disorders distinguishing it from healthy personality as well as from other forms of mental disorders, thus demarcating the “genus of personality pathology” (9). ICD-11 proposes to evaluate the severity of the impairment in personality functioning from no personality pathology through the subsyndromal condition of “personality difficulty” to mild, moderate and severe personality disorder diagnoses. The individual differences between personality disorders (“species”) are then further defined by pathological personality traits. One of the central aims of the new system is to increase clinical utility with the assessment of severity of impairment informing clinical prognosis and treatment intensity, while specific pathological personality traits might help to inform the focus of treatment efforts (10).

This article focuses on a discussion of the newly introduced severity measurement of the ICD-11 classification of personality disorder from a psychodynamic viewpoint and compares it with established psychodynamic assessment instruments and their underlying concepts. A comprehensive analysis of the advantages and limitations of the complete ICD-11 system including a discussion of the complex issue of pathological personality traits is beyond the scope of this article.

## PSychoanalytic Perspectives

The focus on self and interpersonal functioning as the central tenet of personality pathology is shared by many theoretical orientations and evidence-based treatments for personality disorders (9). Notably, this approach converges with long-standing conceptualizations of personality psychology offered by the psychoanalytic/psychodynamic tradition. In a certain sense, the proposition of self- and interpersonal functioning as core features of personality pathology constitutes a re-introduction of psychoanalytic considerations into the diagnostic systems after the “Neo-Kraepelian revolution” associated with the arrival of DSM-III (11).

Personality functioning as defined in the ICD-11 and DSM-5 is related to the psychoanalytic notion of personality structure or organization (these terms are often used synonymously in the psychoanalytic tradition). Personality structure or organization are theoretical concepts characterizing the fundamental “operating system” or underlying, stable configuration of personality, which in turn manifests itself in specific modes of personality functioning which can be phenomenologically observed and assessed. The precursors of the modern psychoanalytic understanding of personality structure can be found in Freud's “structural theory” of the psyche as composed of Id, Ego, and Super-ego and the analysis of the interaction between these entities in psychic functioning (12). Another genealogy links the contemporary concept with the notion of “character neurosis” (cf. Abraham, Reich) as opposed to “symptom neurosis” and the concomitant focus on underlying personality configurations instead of only analyzing specific symptomatic manifestations (13).

Arguably, the most important contemporary psychoanalytic model of personality structure was developed by Otto Kernberg et al. over the past decades (14–16). Kernberg's object-relation model of personality organization integrates findings from developmental psychology, attachment theory, neurobiology as well as classic psychoanalytic clinical theory (17). Early experiences of interactions between infant and caregiver, which are charged by intensive affects, are gradually internalized as “object-relations dyads” of self- and other representations and provide the fundamental building blocks of personality organization. Initially, these object-relation dyads are undifferentiated (clear demarcations between self and other are not yet established) and split between ideally good and persecutory bad experiences. In normal development they gradually become more differentiated and integrated corresponding to the formation of a healthy personality structure including a stable personal identity and a capacity for emotionally satisfying reciprocal object relations. Pathological psychic functioning as, e.g., observed in borderline personality organization corresponds to the presence of unintegrated and split object-relation dyads leading to identity disturbances (identity diffusion) and dysfunctional interpersonal relations (18).

Kernberg's model constitutes one of the cornerstones of the Psychodynamic Diagnostic Manual (PDM-2), a collaborative effort for the comprehensive, psychodynamically informed assessment of personality, (19–21). The PDM-2 gives due weight to empirical as well as the rich clinical knowledge gathered during the last decades and aims to supplement the primarily descriptive psychiatric nosologies in terms of combining nomothetic and idiographic approaches. Specifically, the PDM-2 for adults suggests to assess an individual's (1) level of personality organization and (2) the specific personality style or type. While these organizing principles broadly correspond to the ICD-11 proposal, there are important differences on a deeper level (11, 20). Many of these issues concern the contention that the reliance on isolated symptoms for diagnosis or the use of personality trait models derived from academic psychology do not provide a clinically meaningful description of the personality problems faced by practicing therapists and thus the PDM-2 intends to expand the clinical utility *via* an assessment of personality styles based on underlying dynamic themes and conflicts (19, 22, 23). Regarding the focus of this article, severity of personality disturbance, the PDM-2 closely follows Kernberg's model in differentiating between a normal, neurotic, borderline, and psychotic level of personality organization (19).

In the German-speaking world, the most important psychodynamic model for the assessment of personality structure is provided by the Operationalized Psychodynamic Diagnosis (OPD-2) (24). Conceptually, the OPD model is rooted in psychoanalytic theory (mainly ego-psychological perspectives) as well as in attachment theory and developmental psychology. It represents a comprehensive diagnostic system based on a clinical interview and contains five axes, of which one is dedicated to the assessment of personality structure and covers very similar aspects of personality functioning as ICD-11 and DSM-5. The OPD has been developed from a purely diagnostic system to include a set of tools and procedures for treatment planning and for measuring change, as well as for determining the appropriate main focuses of treatment and developing suitable treatment strategies.

## Discussion of Central Aspects of the ICD-11 Personality Disorder Classification

### Domains of Functioning

There is considerable overlap between the domains of personality functioning deemed relevant by the ICD-11 and psychoanalytic conceptualizations of personality. Table 1 shows the domains and subdomains capturing the central aspects of self- and interpersonal functioning in ICD-11, DSM-5, object relations theory and the OPD-2. Notably, the centrality of disturbances in the area of identity for personality pathology has been a hallmark of Kernberg's system from the 1960s, building upon the foundational work of Erik Erikson and Edith Jacobson. The syndrome of identity diffusion with the persistence of an unintegrated view of oneself and others and a corresponding split between idealized and persercutory internalized experiences with others represents the most important indicator of the severity of a personality disorder (25).

**Table 1:** Domains of self- and interpersonal functioning in ICD-11, DSM-5, object relations theory (STIPO) and OPD.

**ICD-11**		**DSM-5**		**STIPO**		**OPD**	
**Domain**	**Sub-domain**	**Domain**	**Sub-domain**	**Domain**	**Sub-domain**	**Domain**	**Sub-domain**
Self-functioning	Identity Self-worth Accuracy of self-view Self-direction	Self-functioning	Identity Self-direction	Identity	Capacity to invest in work/studies and recreation Sense of self Sense of others	Self	Self-perception Self-regulation Internal communication Attachment to internal objects
Interpersonal functioning	Interest in engaging in relationships with others Ability to develop and maintain close and mutually satisfying relationships Ability to understand others' perspectives Ability to manage conflict in relationships	Interpersonal Functioning	Empathy Intimacy	Object relations	Interpersonal relations Intimate relationships and sexuality Internal working model of relationships	Object	Object-perception Regulation of object relationship Communication with the external world Attachment to external objects

Besides considering the domains of self- and interpersonal functioning for the evaluation of personality disorder severity, the ICD-11 also takes into account emotional, cognitive, and behavioral manifestations of personality dysfunction. This includes the “Accuracy of situational and interpersonal appraisals, especially under stress,” which ranges from adequate reality testing to dissociative or psychotic-like perceptions and beliefs in the most severe cases. The capacity for reality testing also constitutes a core element of the level of personality organization as developed by Kernberg, with impairment in reality testing being the hallmark of a psychotic functioning, and a propensity for paranoid reaction with otherwise intact reality testing being indicative of borderline personality organization (14, 26, 27). From an object-relations perspective, impairment in reality testing corresponds to a refusion of self and object representations leading to de-differentiation and confusion between self and non-self as well as psychic and external reality (28). Arguably, this inclusion in ICD-11 of reality testing as a central dimension of personality disorder severity is more aligned with psychodynamic conceptualizations than the AMPD model of DSM-5, which aims to assess impairment in reality testing with the “Psychoticism” trait.

Despite the significant similarities there are also noteworthy differences. Generally, the psychodynamic models encompass a broader definition of personality functioning and consider several further dimensions essential for the accurate appraisal of personality organization. Some of these domains of psychodynamic personality functioning are evaluated in the trait section of ICD-11, including aggression against self and others. Other dimensions are not represented in the ICD-11, notably including the predominant quality of defense mechanisms and the level of moral functioning. The mechanisms used by an individual to defend against unbearable feelings and mental states range from mature and adaptive processes like intellectualization and repression to primitive and maladaptive ones such as splitting and projective identification. These immature mechanisms are deemed pathognomonic for personality pathology and provide clinically relevant information for appropriate treatment planning and therapeutic technique (29, 30). Additionally, an individual's capacity for guilt and adherence to common norms of interpersonal behavior in the sense of moral functioning is considered to be another key domain of personality functioning. Consistent and integrated moral functioning is a characteristic of normal personality functioning, while distortions and deficits such as lying, stealing or other antisocial behavior is associated with impairment in personality organization. Again, the assessment of moral functioning is crucial for differential treatment planning and prognostic considerations (31).

Another remarkable facet is the almost complete absence of any mention of sexuality in the ICD-11 guidelines. From a psychoanalytic point of view, sexuality constitutes a highly relevant dimension of object relations/interpersonal functioning and difficulties in the establishing mutually gratifying sexual relationships are considered to be core features of personality pathology and should be part of a comprehensive assessment. The ability to integrate intimacy and sexuality in a trusting relationship is one of the cornerstones of a healthy personality (32).

### Atheoretical Description vs. Systematic Theoretical Background

One of the key differences between a psychoanalytic approach to personality disorder assessment and the ICD-11 proposal is that the first one is grounded in a comprehensive theoretical system as opposed to the atheoretical, predominantly descriptive ICD-11 system (33, 34). Object relations theory incorporates findings from psychoanalytic clinical experience, developmental psychology, as well as neurobiology and integrates them into a complex and unified theoretical model of healthy and pathological personality functioning (17, 31). Therefore, object relations theory is not only able to identify the different domains that are shown to be relevant for diagnostic and treatment purposes, but also to provide a framework to understand the relations and links between these different domains. This is of special significance with regard to the crucial question of how to meaningfully integrate the severity rating of the ICD-11 with the proposed trait dimensions, but this issue is beyond the scope of the present article focusing on personality functioning. But also for the core components of the new classification system, a psychoanalytic viewpoint is able to contribute to a deeper understanding. For object relations theory, the development of capacities for self- and interpersonal functioning are inextricably intertwined, since both are rooted in the early experiences of interactions between self and other empowered by strong negative or positive affects. Therefore, the inclusion of the domain of self-functioning in the final version of the ICD-11 personality disorder classification was a definitive improvement over the first proposal of the ICD-11 working group, which exclusively focused on interpersonal–social dysfunction as the core of personality disturbance (7, 35, 36).

### Severity

From a psychodynamic viewpoint, the inclusion of severity of impairment as the core feature of personality disorder classification represents a major improvement over previous classification systems. Multiple studies have confirmed global severity as the single most important predictor for current and future dysfunction (37–39). The consideration of the severity of personality pathology also has been a central component of psychodynamic approaches to personality pathology for decades. Kernberg was one of the first to present a systematic conceptualization of personality disorders located on a spectrum of severity differentiating between four broad levels of personality organization: normal; neurotic; borderline; psychotic (15). Each level of personality organization is characterized by specific impairments in the above mentioned domains. The more recent OPD-2 follows a similar structure and defines seven levels of impairment in personality functioning.

The general description of the different levels of personality functioning are very similar between the ICD-11 system and psychodynamic diagnostic approaches providing convergent validity to the model. However, as noted above, the ICD-11 system provides a purely descriptive approach and in line with its overarching goals does not include any information of etiology and pathogenesis for the different levels of personality pathology. It has been suggested that this atheoretical approach leads to an overemphasis on readily observable behavioral symptoms while somewhat neglecting the intrapsychic dimension which psychodynamic approaches deem crucial for personality pathology. Compared to the DSM-5, it has been noted that the ICD-11 personality classification system predominantly focuses on externalizing behavior and the “dangerous patient” as a criterion for the assessment of personality pathology (40, 41). Arguably, this could in turn lead to an unintended increase in stigmatization associated with a diagnosis of severe personality disorder due to the association of personality pathology with violence and dangerousness. Furthermore, without careful and detailed examination of the internal psychic functioning of the patient there is a danger of a superficial use of a global impression to assess the level of personality pathology severity; patients with a predominantly internalizing personality type might be assessed better than they actually are.

Psychodynamic approaches consider the intrapsychic dimension an irreducible aspect of personality functioning which is reflected in the detailed assessment strategies of the presented models. They each provide a theoretical framework for understanding the developmental pathways leading to the different levels of personality organization and thus go beyond a purely descriptive approach. Notably, object relations theory sees Borderline personality organization as the outcome of the predominance of excessive splitting mechanisms, which impede the development of an integrated view of oneself and significant others, leading to the observable deficits in self- and interpersonal functioning. The predominance of these splitting mechanisms is in turn associated with constitutive (genetic) factors linked to excessive levels of aggression and to environmental containment deficits of primary attachment figures. In normal development, splitting mechanisms gradually diminish and more mature modes of defense mechanisms (e.g., repression) take center stage leading to increased identity integration (25). Therefore, Kernberg's levels of personality organization are not only descriptive categories of symptom severity, but also designate qualitatively distinct modes of psychic functioning. This understanding contains important information for differential treatment planning purposes and guides clinical psychotherapeutic work with patients (15, 31).

### Clinical Utility

One of the central purposes of a diagnostic classification system is to inform clinical decision making including risk assessment and differential treatment planning (42). The ICD and DSM classification systems with their focus on reliability and generalizability have frequently been contested for their limited meaningfulness to the treating clinician, especially in the field of personality pathology (21, 43, 44). The focus on the severity of self- and interpersonal dysfunction represents a considerable step forward in making personality disorder diagnoses clinically meaningful (37). Bach and Simonsen (45) have recently shown that the ICD-11 system can provide a conceptual umbrella for various treatment models targeting key areas of personality functioning, including Schema-Focused Therapy, Mentalization-Based Therapy, and Transference-focused Psychotherapy. Generally, patients with more severe disturbances in personality functioning require more highly structured treatment settings and frequently the establishment of a formal treatment contract is necessary in order to minimize destructive attacks against self, others and the treatment. Furthermore, therapists often need to incorporate supportive elements in their treatment approach in order to counterbalance deficits in personality functioning of the patients. Major treatment objectives include control and resolution of destructive behavior and over time greater stability in self and interpersonal functioning. On the other hand, patients with less severe personality disturbance are able to benefit from less structured treatment modalities and therapists can more readily focus on explorative interventions such as confrontations and interpretations. There is little risk for destructive acting out and treatment goals include a greater depth of experience of self and others as well as more flexible functioning (31, 33, 45).

However, ICD-11 remains at its core an atheoretical (or pantheoretical) and predominantly descriptive diagnostic system, therefore arguably reducing its immediate clinical utility (21). In contrast, the assessment of the severity of impairment in personality functioning has been a cornerstone of psychodynamic diagnostic considerations with clear implications for treatment planning. Object relations theory provides an integrated system for diagnosis, treatment planning, as well as the therapeutic process itself. Transference-Focused Psychotherapy (TFP), the therapeutic approach developed by Kernberg et al. in the last decades (46), is intrinsically related to the diagnostic considerations and to the developmental model outline above. TFP specifically targets deficits in self- and interpersonal functioning by focusing on the analysis of currently activated dyads between therapist and patient and the predominant affect by means of clarification, confrontation and interpretation, thereby enhancing the capacity for reflective functioning and identity integration (31, 46).

Furthermore, the more comprehensive nature of the dimensions of personality functioning assessed in object relations theory and OPD consequently provide more extensive clinically relevant information. For example, the level of moral functioning is considered to be a crucial factor for prognosis and treatment choice with antisocial personality functioning indicating a particularly poor prognosis. Equally, the predominance of immature defense mechanisms will have an impact on all stages of treatment and its assessment will help the clinician to better understand the often rapidly shifting and apparently chaotic behavior of patients suffering from severe personality disorder.

Notably, the assessment of the severity of personality disturbance constitutes only one step of the ICD-11 diagnostic procedure, and careful consideration needs to be given to the aptitude of the supplementing trait model to provide a clinically meaningful picture of the individual personality style or type of a patient (10, 47). Many psychodynamically oriented researchers and practitioners are critical of the ability of trait-based models mainly derived from self-report questionnaires to reflect the complexity and functional interrelatedness of psychological characteristics and the crucial importance of the experiential dimension of meaning for an adequate and clinically valuable assessment of an individual patient (20, 33, 48).

Of course, the most important objection to a more comprehensive model of personality functioning is the limited time available in daily clinical practice. This was a major focus of the ICD-11 working group in developing the new system and it certainly is a crucial factor (1). But the necessity for easy and time-saving diagnostic systems needs to be balanced against the danger of superficial or uncritical use of categories and labels. This seems especially relevant in the field of personality pathology, where careful assessment and the often difficult differential diagnosis require an adequate amount of time and training. Furthermore, patients with personality disorders frequently evoke strong countertransference reactions in others and while these communications can provide invaluable information to the clinician, there is a danger that these emotions are disavowed and unconsciously influence the diagnostic procedure, if not adequately noticed and reflected (49).

### Assessment Instruments

While a number of self-report and interview-based instruments have already been developed for the AMPD of DSM-5 (6), no official instrument to assess the level of impairment in self- and interpersonal functioning according to ICD-11 has been published up to now, which complicates the evaluation of the proposed system. The Standardized Assessment of Severity of Personality Disorder (SASPD), a brief self-report questionnaire (9 items), was developed as a screening tool for an earlier draft of the ICD-11 personality disorders model, but is now outdated with the arrival of the final model (50). New measures for the official new system are currently developed and until then the use of established psychodynamic instruments can provide a pragmatic solution.

Both psychodynamic approaches covered here, object relations theory and OPD, provide both, self-report and interviewer-based assessment instruments. The Structured Interview for Personality Organization [STIPO; (51); Revised version, STIPO-R: (52)] is the structured version for research purposes of the clinical Structural Interview originally developed by Kernberg to assess the level of personality organization (53). In its revised version it consists of 55 items and covers five domains (see Table 2). The evaluation of the domains yields an overall rating of five different levels of personality organization (i.e., personality functioning) (1) normal, (2) neurotic; (3) borderline 1, (4) borderline 2, and (5) borderline 3. There also exists a self-report measure (57 items), the Inventory of Personality Organization [IPO; (26)] as well as a 16-item short version of the questionnaire [IPO-16; (54)].

**Table 2:** STIPO-R domains and subdomains.

**Domain**	**Subdomain**
Identity	Capacity to invest in work/studies and recreation
	Sense of self
	Sense of others
Object relations	Interpersonal relations
	Intimate relationships and sexuality
	Internal working model of relationships
Defenses	Lower-level, primitive defenses
	Higher-level defenses
Aggression	Self-directed aggression
	Other-directed aggression
Moral values	Experience of guilt
	Moral and immoral behavior

The Operationalized Psychodynamic Diagnosis (OPD-2) (24) represents a comprehensive diagnostic system and contains five axes: (I) experience of illness and prerequisites for treatment, (II) interpersonal relations, (III) conflict, (IV) structure, and (V) mental and psychosomatic disorders (according to ICD or DSM). The rating is performed after a partly structured and predominantly unstructured psychodynamic initial interview. The OPD-2 structure axis covers eight domains with 3 structural facets each (see Table 3). As a diagnostic instrument, the OPD-2 structure axis has been used in a large number of empirical studies involving personality functioning and has demonstrated good reliability as well as concurrent and discriminant validity (55, 56) and can thus be recommended for clinical use and research purposes. For research purposes, a video-taping of the interview and a rating by two independent raters is demanded. The clinical rating can be done by the interviewer him-/herself; compared to more structured interview approaches, the OPD-2 interview reveals a lot of clinical information beyond the quantifying rating, that is useful for treatment planning. Moreover, the assessment of all five axes mentioned above allows for a complete case formulation (24, 57). The full clinical OPD interview takes 1–2 h. A corresponding 95-item questionnaire (OPD-SQ) has been developed and validated (58) including a one-dimensional short-form of 12 items [OPD-SQS; (59)].

**Table 3:** OPD-2 structural domains and facets.

**Self**	**Object**
Cognitive ability: self-perception Self-reflection Affect differentiation Identity	Cognitive ability: object perception Self/object differentiation Whole object perception Realistic object perception
Capacity for regulation: self-regulation Impulse control Affect tolerance Self-worth regulation	Capacity for regulation: regulation of object relationship Protecting relationships Balancing of interests Anticipation
Emotional ability: internal communication Experiencing affects Use of fantasies Bodily self	Emotional ability: communication with the external world Making contact Communication of affect Empathy
Attachment capacity: internal objects Internalization Use of introjects Variable attachments	Attachment capacity: external objects Ability to make attachments Accepting help Severing attachments

Due to the scarcity of published instruments specifically designed for the ICD-11, there is only limited direct empirical evidence available on the validity and utility of the ICD-11 definition of personality functioning to date. The—now outdated—SASPD has shown good predictive ability for the presence of mild or moderate personality pathology severity with limited ability to discriminate severe personality pathology (50). A number of studies empirically compared ICD-11 (SASPD) and DSM-5 (Level of Personality Functioning Scale, LPFS) (40, 41, 60), generally showing good convergence between these measures of personality pathology severity. But the results also show that the SASPD predominantly captures externalizing, interpersonal difficulties, while somewhat neglecting the area of self-functioning and internal distress. Additionally, Zimmermann et al. (39) raised concern about the reliability of the SASPD to measure personality pathology severity when compared to 5 other self-report instruments derived from DSM-5 (LPFS—Brief Form 2.0, LPFS—Self Report, Personality Inventory for DSM-5—Brief Form Plus) and the psychodynamic tradition (IPO-16, OPD-SQS), arguably equally due to its lack of incorporation of items assessing the self-related dimension of personality functioning. Accordingly, the authors found a stronger correlation between the psychodynamic instruments and the AMPD of DSM-5 than with the ICD-11 system (39). Similar results were provided by Oltmanns and Widiger, who found a significantly higher correlation between the IPO and LPFS than between these two and the SASPD (41).

These findings most likely reflect the specific construction of the SASPD, which was based on an earlier draft version of the ICD-11 personality disorders classification and are therefore not representative of the final official ICD-11 system. Therefore, the development of carefully designed new instruments to adequately capture all facets of the official new system is essential [Bach and Simonsen recently reported significant advances on this topic (45)]. Especially, there is a need for expert-rated instruments and (semi-)structured interviews of personality functioning according to the ICD-11 framework in order to confront the well-known challenges of self-report instruments in personality disorder research.

## Conclusion

The influence of contemporary psychoanalytic object-relations theory, most prominently advocated by Kernberg et al., on the development on the AMPD of DSM-5 has been frequently highlighted (37). While this influence is not explicitly stated in the reports of the ICD-11 working group for the reclassification of personality disorders, the final version of the classification system is highly compatible with a psychoanalytic framework of personality pathology (45), but the focus on self and interpersonal functioning for the assessment of personality pathology constitutes a pantheoretical approach and is not limited to the psychoanalytic tradition. Arguably, personality functioning can be considered to be the current “common ground” for the assessment of personality disorders and therefore constitutes a considerable step forward in the effort to provide a foundation for a field with a long history of rivaling schools of thought often deemed to be incommensurable.

## Author Contributions

VB and SD conceptualized the manuscript. VB wrote the first draft of the manuscript. SD substantially revised the initial draft. Both authors contributed to the article and approved the submitted version.

## Conflict of Interest

The authors declare that the research was conducted in the absence of any commercial or financial relationships that could be construed as a potential conflict of interest.

## References

[1] Tyrer P Mulder R Kim YR Crawford MJ. The development of the ICD-11 classification of personality disorders: an amalgam of science, pragmatism, and politics. Annu Rev Clin Psychol. (2019) 15:481–502. 10.1146/annurev-clinpsy-050718-095736 30601688

[2] Mulder R Tyrer P. Diagnosis and classification of personality disorders: novel approaches. Curr Opin Psychiatry. (2019) 32:27–31. 10.1097/YCO.0000000000000461 30234525

[3] Widiger TA Samuel DB. Diagnostic categories or dimensions? A question for the diagnostic and statistical manual of mental disorders–fifth edition. J Abnorm Psychol. (2005) 114:494–504. 10.1037/0021-843X.114.4.494 16351373

[4] Skodol AE Morey LC Bender DS Oldham JM. The ironic fate of the personality disorders in DSM-5. Personal Disord. (2013) 4:342–9. 10.1037/per0000029 24378161

[5] Association AP. Diagnostic and Statistical Manual of Mental Disorders: DSM-5. 5th ed. Washington, DC: American Psychiatric Association (2013). p. 947.

[6] Zimmermann J Kerber A Rek K Hopwood CJ Krueger RF. A Brief but comprehensive review of research on the alternative DSM-5 model for personality disorders. Curr Psychiatry Rep. (2019) 21:92. 10.1007/s11920-019-1079-z 31410586

[7] Herpertz SC Huprich SK Bohus M Chanen A Goodman M Mehlum L. The challenge of transforming the diagnostic system of personality disorders. J Pers Disord. (2017) 31:577–89. 10.1521/pedi_2017_31_338 28910213 PMC5735999

[8] World Health Organization. International Classification of Disesases. 11th ed. (ICD-11). World Health Organization.

[9] Pincus AL Cain NM Halberstadt AL. Importance of self and other in defining personality pathology. Psychopathology. (2020) 53:133–40. 10.1159/000506313 32114579

[10] Bach B First MB. Application of the ICD-11 classification of personality disorders. BMC Psychiatry. (2018) 18:351. 10.1186/s12888-018-1908-3 30373564 PMC6206910

[11] Natoli AP. The DSM's reconnection to psychoanalytic theory through the alternative model for personality disorders. J Am Psychoanal Ass. (2019) 67:1023–45. 10.1177/0003065120903060 32043386

[12] Freud S. The ego and the Id. In: Strachey J editor. The Standard Edition of the Complete Psychological Works of Sigmund Freud. Vol. 19. London: Hogarth Press (1923).

[13] McWilliams N. Psychoanalytic Diagnosis: Understanding Personality Structure in the Clinical Process. 2nd ed. New York: Guilford Press (2011). p. 426.

[14] Kernberg O. Borderline personality organization. J Am Psychoanal Assoc. (1967) 15:641–85. 10.1177/000306516701500309 4861171

[15] Kernberg OF. Severe Personality Disorders: Psychotherapeutic Strategies. New Haven: Yale University Press (1984). p. 381.

[16] Kernberg O. Structural derivatives of object relationships. Int J Psychoanal. (1966) 47:236–53. 5964140

[17] Kernberg OF Caligor E. A psychoanalytic theory of personality disorders. In: Lenzenweger MF Clarkin JF editors. Major Theories of Personality Disorder. 2nd ed. New York, NY: Guilford Press (2005). p. 114–56.

[18] Clarkin JF Lenzenweger MF Yeomans F Levy KN Kernberg OF. An object relations model of borderline pathology. J Pers Disord. (2007) 21:474–99. 10.1521/pedi.2007.21.5.474 17953502

[19] Lingiardi V McWilliams N. Psychodynamic Diagnostic Manual: PDM-2. 2nd ed. New York, NY: The Guilford Press (2017). p. 1078.

[20] McWilliams N Grenyer BFS Shedler J. Personality in PDM-2: controversial issues. Psychoanal Psychol. (2018) 35:299–305. 10.1037/pap0000198

[21] Lingiardi V McWilliams N Bornstein RF Gazzillo F Gordon RM. The psychodynamic diagnostic manual version 2 (PDM-2): assessing patients for improved clinical practice and research. Psychoanal Psychol. (2015) 32:94. 10.1037/a0038546

[22] McWilliams N. Beyond traits: personality as intersubjective themes. J Pers Assess. (2012) 94:563–70. 10.1080/00223891.2012.711790 22882014

[23] Westen D Shedler J Bradley B DeFife JA. An empirically derived taxonomy for personality diagnosis: bridging science and practice in conceptualizing personality. Am J Psychiatry. (2012) 169:273–84. 10.1176/appi.ajp.2011.11020274 22193534 PMC4546840

[24] Force OT. Operationalized Psychodynamic Diagnosis OPD-2: Manual of Diagnosis and Treatment Planning. Göttingen: Hogrefe Publishing (2008).

[25] Kernberg OF. Identity: recent findings and clinical implications. Psychoanal Q. (2006) 75:969–1004. 10.1002/j.2167-4086.2006.tb00065.x 17094369

[26] Lenzenweger MF Clarkin JF Kernberg OF Foelsch PA. The inventory of personality organization: psychometric properties, factorial composition, and criterion relations with affect, aggressive dyscontrol, psychosis proneness, and self-domains in a nonclinical sample. Psychol Assess. (2001) 13:577–91. 10.1037/1040-3590.13.4.577 11793901

[27] Dagnall N Denovan A Parker A Drinkwater K Walsh RS. Confirmatory factor analysis of the inventory of personality organization-reality testing subscale. Front Psychol. (2018) 9:1116. 10.3389/fpsyg.2018.01116 30026714 PMC6041939

[28] Kernberg OF. Psychotic personality structure. Psychodyn Psychiatry. (2019) 47:353–72. 10.1521/pdps.2019.47.4.353 31913791

[29] Leichsenring F Kunst H Hoyer J. Borderline personality organization in violent offenders: correlations of identity diffusion and primitive defense mechanisms with antisocial features, neuroticism, and interpersonal problems. Bull Menninger Clin. (2003) 67:314–27. 10.1521/bumc.67.4.314.26983 14733448

[30] Kernberg OF Yeomans FE Clarkin JF Levy KN. Transference focused psychotherapy: overview and update. Int J Psychoanal. (2008) 89:601–20. 10.1111/j.1745-8315.2008.00046.x 18558958

[31] Caligor E Kernberg OF Clarkin JF Yeomans FE American Psychiatric Association Publishing. Psychodynamic Therapy for Personality Pathology: Treating Self and Interpersonal Functioning. Washington, DC: American Psychiatric Association Publishing (2018).

[32] Kernberg OF. Love Relations: Normality and Pathology. London: Yale University Press (1998).

[33] Clarkin JF Caligor E Sowislo JF. An Object relations model perspective on the alternative model for personality disorders (DSM-5). Psychopathology. (2020) 53:141–8. 10.1159/000508353 32698184 PMC7949219

[34] Follette WC Houts AC. Models of scientific progress and the role of theory in taxonomy development: a case study of the DSM. J Consult Clin Psychol. (1996) 64:1120–32. 10.1037/0022-006X.64.6.1120 8991299

[35] Tyrer P Crawford M Mulder R Blashfield R Farnam A Fossati A. The rationale for the reclassification of personality disorder in the 11th revision of the International Classification of Diseases (ICD-11). Personal Ment Health. (2011) 5:246–59. 10.1002/pmh.190

[36] Tyrer P Crawford M Mulder R Classi I-WGR. Reclassifying personality disorders. Lancet. (2011) 377:1814–5. 10.1016/S0140-6736(10)61926-5 21353696

[37] Bender DS Morey LC Skodol AE. Toward a model for assessing level of personality functioning in DSM-5, part I: a review of theory and methods. J Pers Assess. (2011) 93:332–46. 10.1080/00223891.2011.583808 22804672

[38] Tyrer P. The problem of severity in the classification of personality disorder. J Pers Disord. (2005) 19:309–14. 10.1521/pedi.2005.19.3.309 16175739

[39] Zimmermann J Muller S Bach B Hutsebaut J Hummelen B Fischer F. A common metric for self-reported severity of personality disorder. Psychopathology. (2020) 53:168–78. 10.1159/000507377

[40] Bach B Anderson JL. Patient-reported ICD-11 personality disorder severity and DSM-5 level of personality functioning. J Pers Disord. (2020) 34:231–49. 10.1521/pedi_2018_32_393 30179575

[41] Oltmanns JR Widiger TA. Evaluating the assessment of the ICD-11 personality disorder diagnostic system. Psychol Assess. (2019) 31:674–84. 10.1037/pas0000693 30628821 PMC6488396

[42] Reed GM First MB Kogan CS Hyman SE Gureje O Gaebel W. Innovations and changes in the ICD-11 classification of mental, behavioural and neurodevelopmental disorders. World Psychiatry. (2019) 18:3–19. 10.1002/wps.20611 30600616 PMC6313247

[43] Morey LC Skodol AE Oldham JM. Clinician judgments of clinical utility: a comparison of DSM-IV-TR personality disorders and the alternative model for DSM-5 personality disorders. J Abnorm Psychol. (2014) 123:398–405. 10.1037/a0036481 24886013

[44] Clarkin JF Huprich SK. Do Dsm-5 personality disorder proposals meet criteria for clinical utility? J Pers Disord. (2011) 25:192–205. 10.1521/pedi.2011.25.2.192 21466249

[45] Bach B Simonsen S. How does level of personality functioning inform clinical management and treatment? Implications for ICD-11 classification of personality disorder severity. Curr Opin Psychiatry. (2021) 34:54–63. 10.1097/YCO.0000000000000658 33252430

[46] Yeomans F Clarkin JF Kernberg OF. Transference-Focused Psychotherapy for Borderline Personality Disorder: A Clinical Guide. Washington, DC: American Psychiatric Publishing (2015). 10.1176/appi.books.9781615371006

[47] Oltmanns JR. Personality traits in the international classification of diseases 11th revision (ICD-11). Curr Opin Psychiatry. (2021) 34:48–53. 10.1097/YCO.0000000000000656 33252429

[48] Huprich SK. Personality disorders in the ICD-11: opportunities and challenges for advancing the diagnosis of personality pathology. Curr Psychiat Rep. (2020) 22:40. 10.1007/s11920-020-01161-4 32519211

[49] Rossberg JI Karterud S Pedersen G Friis S. An empirical study of countertransference reactions toward patients with personality disorders. Compr Psychiatry. (2007) 48:225–30. 10.1016/j.comppsych.2007.02.002 17445515

[50] Olajide K Munjiza J Moran P O'Connell L Newton-Howes G Bassett P. Development and psychometric properties of the standardized assessment of severity of personality disorder (SASPD). J Pers Disord. (2018) 32:44–56. 10.1521/pedi_2017_31_285 28513349

[51] Clarkin JF Caligor E Stern B Kernberg O. Structured Interview for Personality Organization (STIPO). New York, NY: Weill Medical College of Cornell University (2004).

[52] Clarkin J Caligor E Stern B Kernberg O. The Structured Interview of Personality Organization-Revised (STIPO-R). New York, NY (2016).

[53] Stern BL Caligor E Clarkin JF Critchfield KL Horz S MacCornack V. Structured interview of personality organization (STIPO): preliminary psychometrics in a clinical sample. J Pers Assess. (2010) 92:35–44. 10.1080/00223890903379308 20013454

[54] Zimmermann J Benecke C Horz S Rentrop M Peham D Bock A. Validity of a German 16-item version of the inventory of personality organization (IPO-16). Diagnostica. (2013) 59:3–16. 10.1026/0012-1924/a00007625831981

[55] Doering S Burgmer M Heuft G Menke D Baumer B Lubking M. Assessment of personality functioning: validity of the operationalized psychodynamic diagnosis axis IV (structure). Psychopathology. (2014) 47:185–93. 10.1159/000355062 24192300

[56] Zimmermann J Ehrenthal JC Cierpka M Schauenburg H Doering S Benecke C. Assessing the level of structural integration using operationalized psychodynamic diagnosis (OPD): implications for DSM-5. J Pers Assess. (2012) 94:522–32. 10.1080/00223891.2012.700664 22808938

[57] Ehrenthal JC Benecke C. Tailored treatment planning for individuals with personality disorders: the operationalized psychodynamic diagnosis (OPD) approach. In: Kramer U editor. Case Formulation for Personality Disorders. Cambridge: Academic Press (2019). p. 291–314. 10.1016/B978-0-12-813521-1.00015-1

[58] Ehrenthal JC Dinger U Horsch L Komo-Lang M Klinkerfuss M Grande T. The OPD structure questionnaire (OPD-SQ): first results on reliability and validity. Psychother Psych Med. (2012) 62:25–32. 10.1055/s-0031-1295481 22271173

[59] Ehrenthal JC Dinger U Schauenburg H Horsch L Dahlbender RW Gierk B. Development of a 12-item version of the OPD-Structure Questionnaire (OPD-SQS). Z Psychosom Med Psyc. (2015) 61:262–74. 10.13109/zptm.2015.61.3.262 26388057

[60] McCabe GA Widiger TA. A comprehensive comparison of the ICD-11 and DSM-5 section III personality disorder models. Psychol Assess. (2020) 32:72–84. 10.1037/pas0000772 31580095

